# Efficacy of metformin in combination with immune checkpoint inhibitors (anti-PD-1/anti-CTLA-4) in metastatic malignant melanoma

**DOI:** 10.1186/s40425-018-0375-1

**Published:** 2018-07-02

**Authors:** Muhammad Zubair Afzal, Rima R. Mercado, Keisuke Shirai

**Affiliations:** 1 Hospital Medicine, Dartmouth-Hitchcock Medical Center, One Medical Center Drive, Lebanon, NH 03756 USA; 2 Hematology/Oncology, Norris Cotton Cancer Center, One Medical Center Drive, Lebanon, NH 03756 USA

**Keywords:** Malignant melanoma, Metformin, Pembrolizumab, Ipilimumab, Nivolumab, Anti-PD-1/anti-CTLA-4

## Abstract

**Background:**

Metformin is one of the biguanides commonly used in patients with type II Diabetes Mellitus. Apart from its hypoglycemic properties, metformin also inhibits the cell cycle by restricting protein synthesis and cell proliferation via regulating the LKB1/AMPL pathway. Furthermore, it also enhances the PD-1 blockade through a reduction of tumor hypoxia. Metformin has shown a significant favorable impact on treatment-related outcomes in solid tumors, but these outcomes have not been replicated in the limited clinical studies done on malignant melanoma. Moreover, none of these studies have reported on the efficacy of the combined use of metformin and immune checkpoint inhibitors (ICIs).

**Methods:**

This is a retrospective cohort study that includes patients diagnosed with metastatic malignant melanoma and treated with ipilimumab, nivolumab, and/or pembrolizumab (Cohort A); or ipilimumab, nivolumab, and/or pembrolizumab plus metformin (Cohort B) between January 1st 2011 through December 15th 2017. In this study, patients are stratified based on anti-PD-1 only and anti-CTLA4/anti-PD-1 combination therapies in each cohort. Objective response rate (ORR) is the primary endpoint. Disease control rate (DCR), overall survival (OS) and progression-free survival (PFS) are the secondary endpoints.

**Results:**

Cohort A had 33 patients (60%), while cohort B had 22 (40%). Overall patient characteristics were similar between both cohorts. ORR was higher in cohort B (68.2% vs. 54.5%, *P* = 0.31). The DCR was higher in cohort B as well (77.3% vs. 60.6%, *P* = 0.19). Median OS (46.7 months vs. 28 months), and median PFS (19.8 months vs. 5 months) were longer in cohort B. However, on univariate and multivariate analyses, none of these differences were statistically significant. The mean number of new metastatic sites which appeared during therapy were significantly higher in cohort A (A:1.51 vs. B:0.59, *P* = 0.009).

**Conclusion:**

We have observed favorable treatment-related outcomes (ORR, DCR, median PFS and median OS) in patients who have received metformin in combination with ICIs without reaching significance, probably, due to small sample size. Hence, large prospective clinical trials are required to study the synergistic effect of metformin in combination with ICIs before it can be recommended as routine additive therapy.

## Background

Metformin belongs to the biguanide class of oral hypoglycemic drugs widely used in the treatment of type II Diabetes Mellitus [1]. Metformin increases insulin sensitivity which results in increased glucose uptake and decreased gluconeogenesis, thereby reducing serum glucose levels [1–3]. Metformin inhibits gluconeogenesis from the liver by regulating the adenosine monophosphate-activated protein kinase (AMPK) and liver kinase B1 (LKB1) pathways which inhibit the mammalian target of rapamycin (mTOR). This results in the inhibition of both protein synthesis and gluconeogenesis [3–5]. The LKB1/AMPK pathway is involved in cell cycle regulation by controlling protein synthesis and cell proliferation through modulating the energy required by the cells [6]. This regulation of the LKB1/AMPK pathway inhibits the proliferation of cancer cells and causes apoptosis via an energy deficient stress response [7, 8]. Metformin is also known to inhibit the unfolded protein response (UPR), activate the immune response, and possibly target cancer cells [8]. Since insulin and insulin-like growth factors (IGF1/2) are the key regulators of metabolism, growth, and the cell cycle, metformin exerts an indirect effect on cell growth and proliferation by lowering insulin levels in the body, which it does by reducing IGF and insulin signaling [9]. These hypotheses have been tested on various animal models to study the effect of Metformin on different malignant tissues. In vitro and in vivo studies have shown inhibition of proliferation and delay in the onset of tumor progression in p53 mutant colon cancer mouse models [10, 11]. Furthermore, in vitro studies have demonstrated the inhibition of tumor proliferation in breast, ovarian, and lung cancers [12, 13]. One study has also shown that the routinely used dose of metformin can exert anti-cancer properties [14]. Based on these observations in animal models, various population-based cohort studies have been conducted, which demonstrate the tumor suppressive benefits of metformin in colon, pancreatic, breast, liver, esophageal, gastric, and ovarian cancers, etc. [13].

Malignant melanoma accounts for 5.3% of all new cancer cases and 1.5% of all cancer-related deaths. It has been estimated that 91,270 new cases will be diagnosed in 2018 in the USA alone [15]. Melanoma progression is promoted by epithelial-mesenchymal transition (EMT) that plays a vital role in the radial growth phase (RGP) and invasive vertical growth phase (VGP)—crucial steps in the local invasion and promotion of metastases [16, 17]. Cerezo et al. reported that metformin inhibits the invasion of melanoma cells by regulating the EMT-like factors. In addition, metformin also inhibits the melanoma invasion mediated by AMPK and p53 activation [18]. Tomic et al. reported that metformin induces cell cycle arrest in the G0 and G1 phases and promotes autophagy and apoptosis in different melanoma cells independent of B-RAF and N-RAS mutational status [9, 19]. In the last 10 years, promising targeted therapies have been developed for the treatment of malignant melanoma such as B-RAF inhibitors (vemurafenib, dabrafenib), as well as immunotherapies such as ICIs (ipilimumab, nivolumab, and pembrolizumab). However, the majority of these drugs only have a temporary treatment response due to the emergence of resistance, against B-RAF inhibitors, for instance [20]. Moreover, a long-term follow-up of clinical trial populations receiving ICIs has shown delayed relapse, most likely due to a development of acquired resistance in these patients [21]. These cases of drug resistance and lack of clinical response result in significantly lower overall survival (OS) and life expectancy [22]. One of the mechanisms behind resistance development is the emergence of metabolically modified cells depending mainly on oxidative ATP production [23]. Since metformin induces oxidative stress on tumor cells resulting in cell death [8], it has been suggested that metformin be used in conjunction with targeted therapies to decrease the emergence of resistance [9, 23]. Levingstone et al. compared 10 patients with B-RAF mutant melanoma on metformin plus dabrafenib, with 177 B-RAF mutant melanoma patients on dabrafenib alone. No difference in OS, PFS and RR was observed between these cohorts. However, this study is unfortunately limited by small sample size and low power [23]. Another open-label, prospective pilot study (NCT01840007) had used high dose metformin monotherapy (1000 mg three times a day) in malignant melanoma patients being treated with B-RAF inhibitors or any other first-line chemotherapy, patients who did not respond to ipilimumab, or patients not eligible for ipilimumab therapy. This study was later aborted, as the primary endpoint of increased ORR was not reached [24].

FDA approved ipilimumab in 2011; pembrolizumab and nivolumab in 2014; and ipilimumab-nivolumab combination in 2015 for malignant melanoma. Since the FDA’s approval, these ICIs have been widely used regardless of PD-L1 and B-RAF mutation status. In our review of literature, we have been unable to find any study evaluating the efficacy of metformin when used in combination with ICIs. In one study using mouse models, the combination of anti-PD-1 and metformin was found to have a potential benefit, as it showed that the metformin-induced reduction of tumor hypoxia may enhance the efficacy of PD-1 blockade. In this study, metformin was used to modulate the oxygen tension in the tumor micro-environment. This modulating effect of metformin had a significantly positive impact on the efficacy of the PD-1 blockade [25]. Phenformin, another biguanide, also inhibits the myeloid-derived suppressor cells (MDSCs) and as a result, enhances the anti-tumor activity of PD-1 blockade [26]. These MDSCs are the type of immune cells that promote tumor-induced immune suppression. This immune suppression assists in the evasion of the tumor cells by the immune system [27].

Based on these observations, we aim to study the effect of metformin in patients with malignant melanoma being treated with ICIs simultaneously. To our knowledge, this is the first retrospective evaluation of metformin activity in combination with ICI in literature.

### Study methods

This is a retrospective chart review study conducted at Norris Cotton Cancer and Dartmouth-Hitchcock Medical Centers (DHMC). The DHMC coding department was contacted to identify patients 18 years of age or older who have been diagnosed with advanced malignant melanoma (Stage IIIC, IV) between January 1st 2011 and December 15th 2017, and who had received ipilimumab, nivolumab, and pembrolizumab (or investigational anti-PD-1) without concurrent use of metformin (cohort A), or with concurrent use of metformin (cohort B). Patients taking metformin during the course of ICIs for at least 1 week were included in cohort B. Patients with a history of prior immunotherapy/ICI use, chemotherapy, and previous history of autoimmune disease were not excluded from the study. Due to the retrospective nature of this study, there was no direct patient contact. The institutional review board (IRB) was contacted to obtain an exemption from the informed consent. Patients less than 18 years of age and those whose duration of metformin therapy was less than 1 week were excluded from the study.

Electronic chart reviews were performed. Further data included the basic demographics, TNM stage, mutational status, diabetic status, metformin dose, type of immunotherapy/chemotherapy, radiation therapy, best response (BR)—further classified as complete remission (CR), progressive disease (PD), partial response (PR) and stable disease (SD), as well as time to achieve best radiographic response (RR) were recorded. BR was determined from radiographic images (shrinkage of the lesions on radiographic studies) using the RECIST v. 1.1 criteria [28]. Complete remission (CR) is defined as radiographic disappearance of all target lesions; partial response (PR) is defined as 30% decrease in the target lesions; stable disease (SD) is defined as no significant increase or decrease in the size of the target lesions; and progressive disease (PD) is defined as appearance of any new lesion or an increase in the size of the known lesions by 20% or more [28]. ORR was calculated and defined as the percentage of patients who achieved either PR or CR. DCR was defined as the total percentage of patients achieving CR, PR, and SD. OS and PFS were likewise calculated. OS encompasses the period from the initiation of therapy (ICI ± metformin) until the last follow up date (February 15th 2018) in case the patient is alive, or until the date of death. PFS is defined as there being no objective worsening of the disease while the patient is on therapy (ICI ± metformin), calculated from the date of therapy initiation until the last follow up date (February 15th 2018), or the date of progression of the disease, or the date of death. Eastern Cooperative Oncology Group Performance Status (ECOG-PS) score was obtained from the chart review as well as observed side effects and intolerance (lethargy, weakness, recurrent infections, hospitalizations, withholding of the drug and reason for that). Immune-related adverse events were also recorded. Metastatic sites involved before and during the therapy were documented as well. Finally, the patients were also stratified based on the types of ICI used (anti-PD-1 and anti-PD-1/anti-CTLA-4 combination) in each cohort for comparative analysis.

Independent variables are age, sex, type of mutation, TNM stage, anatomical sites involved and category of therapy (ipilimumab, nivolumab or pembrolizumab with or without metformin). Dependent variables are the best response (BR), ORR, OS, PFS, reported side effects of the therapy, and ECOG score. ORR is the primary endpoint. OS, PFS, DCR are the secondary endpoints.

### Statistical analysis

Due to a small sample size of cohort B (22 patients), no power analysis was performed, and non-probability convenience sampling was done for cohort A. For cohort A, initially, 100 patients with malignant melanoma were identified since 01/01/2011. 35 patients were then randomly selected via a random number generator online tool [29], and 33 of these patients met the inclusion criteria. Summary measures of continuous data such as age at diagnosis, laboratory data, OS, PFS, mean, median, standard deviation (SD), and inter-quartile range were calculated. Histograms and qq-plots of continuous endpoints were used to evaluate distributional assumptions. To evaluate the OS and PFS with 95% CI, Kaplan-Meier method and the log-rank test were applied. Cox regression was applied to calculate the hazard ratio, to take into account other potential confounders such as age at diagnosis, sex, any other malignancy, and prior therapy that may have influenced survival or progression of the disease. Multiple-linear regression and logistic regression analyses were performed to investigate the effect of duration of metformin therapy on OS, disease progression, and PFS. Chi-square and Fisher exact tests were applied to compare the categorical variables and calculate the *P*-value. T-tests were applied to analyze the continuous variables and to calculate the *P*-values.

## Results

### Patient characteristics

Fifty-five patients were included in the final analysis. Thirty-three (60%) of these patients did not receive any metformin (cohort A), while 22 (40%) had received metformin (cohort B). One patient from cohort A had mucosal melanoma. Two patients from cohort A were lost to follow up. Overall mean age at the time of diagnosis of the advanced disease was 63 ± 14.6 years. The mean age at diagnosis was higher in cohort B, although the difference was not statistically significant (67.4 ± 12.6 vs. 60.8 ± 15.3, *P* = 0.08). Cohort A had more female patients and cohort B had more male patients, though the difference was not significant (*P* = 0.42). A higher proportion of patients passed away in cohort A by the last follow up (36.4% vs. 22.7%, *P* = 0.28). Cohort B had higher B-RAF mutations (73.3% vs. 58.3%, *P* = 0.34), and N-RAS mutations were evenly distributed between both cohorts. The mean number of mutations were also similar between both cohorts. TNM stage distribution was similar as well (*P* = 0.23). 100% patients had stage IV melanoma in cohort B vs. 94% in cohort A. Two (6%) patients in cohort A had unresectable stage IIIC disease. An almost equal proportion of patients received anti-PD-1 (pembrolizumab/nivolumab/investigational therapy) and anti-PD-1/anti-CTLA4 combination in both cohorts, while a slightly higher proportion of patients received ipilimumab in cohort A than cohort B (12.1% vs. 9.1%). An equal proportion of the patients received prior immunotherapy and/or chemotherapy before starting the therapy of interest in both cohorts [14 (42.4%) vs. 9 (40.1%) respectively, *P* = 0.9], and a slightly higher proportion of patients received radiation therapy in cohort A (Table 1). B-RAF/MEK inhibitors, interferon, IL-2, alkylating agents, and vascular endothelial growth factor A (VEGF-A) are the additional agents used in both cohorts.

**Table 1:** General Patient Characteristics (IAE’s = Immune-related Adverse Events)

Variables	Cohort A (No Metformin) *N* = 33 (60%)	Cohort B (Metformin) *N* = 22 (40%)	
Mean Age at Diagnosis (Years)	60.8 ± 15.3	67.4 ± 12.6	*P* = 0.08
Sex			
Male	19 (57.6%)	15 (68.2%)	*P* = 0.42
Female	14 (42.4%)	7 (31.8%)	
Dead	11 (36.4%)	5 (22.7%)	*P* = 0.28
TNM Stage			
IIIC	2 (6%)	0 (0%)	
IV	31 (94%)	22 (100%)	*P* = 0.23
BRAF Mutations	14 (58.3%)	11 (73.3%)	*P* = 0.34
NRAS Mutations	5 (21.7%)	4 (26.7%)	*P* = 0.67
Mean no. of mutations	1.3 ± 0.8	1.46 ± 0.9	*P* = 0.67
History of Diabetes	1 (3%)	17 (77.3%)	*P* < 0.001
Median Metformin Dose		500 mg BID (Range 500 mg BID – 1000 mg BID)	
Median duration of Metformin Therapy (Months)		13.5 (0.5–108.7)	
Radiation therapy	6 (18.2%)	2 (9%)	*P* = 0.34
Prior Chemo/Immunotherapy	14 (42.4%)	9 (40.9%)	*P* = 0.91
Immunotherapy			
Anti- PD-1 (Pembrolizumab, Nivolumab)	20 (60.6%)	14 (63.6%)	*P* = 0.93
Ipilimumab/Nivolumab	9 (27.2%)	6 (27.3%)	
Ipilimumab	4 (12.1%)	2 (9.1%)	
Progression	20 (60.6%)	10 (45.4%)	*P* = 0.26
Mean number of metastatic site involved before starting therapy	2.54 ± 1	2.36± 0.95	*P* = 0.5
Mean number of new metastatic site appeared while on therapy	1.51±1.5	0.59± 0.5	*P* = 0.009
Skeletal Metastasis			
Before therapy	7 (21.2%)	4 (18.2%)	*P* = 0.78
During Therapy	6 (18.2%)	3 (13.6%)	*P* = 0.69
Brain Metastasis			
Before therapy	6 (18.2%)	2 (9%)	*P* = 0.34
New brain metastasis during therapy	5 (15.2%)	1 (4.5%)	*P* = 0.21
IAE	20 (60%)	13 (59%)	*P* = 0.91
Prednisone required for IAE’s	18 (54.5%)	13 (59%)	*P* = 0.73
Performance Status			
0	12 (36.4%)	11 (50%)	*P* = 0.33
1	14 (42.4%)	8 (36.4%)	
2	7 (21.2%)	2 (9.1%)	
3	0 (0%)	1 (4.5%)	

Seventeen (77.3%) patients had diabetes in cohort B compared to only one (4.3%) patient in cohort A (*P* < 0.001). Four (18.2%) patients received metformin for steroid-induced hyperglycemia, and in 1 (4.5%) patient metformin was because of its anti-tumor properties and that the patient was gaining weight. The dose of metformin in these patients was 500 mg twice daily, and the median duration of metformin therapy in such patients was 1.5 months. The patient who received metformin for its anti-tumor properties/weight gain, received it for only 2 weeks. Overall, the median duration of metformin therapy was 13.5 months. In majority of the patients, the metformin dose was 500 mg twice daily. The range of metformin dose was 500 mg to 1000 mg twice daily. (Table 1).

The mean number of metastatic sites before initiating the therapy of interest in both cohorts were similar (2.54 vs. 2.36, *P* = 0.5). However, the mean number of new metastatic sites that appeared on distant organs while patients were on therapy was significantly higher in cohort A (A:1.51 vs. B:0.59, *P* = 0.009).The number of skeletal metastases before therapy were slightly higher in cohort A. The appearance of new brain metastases while on therapy was higher in cohort A as well (Table 1). The brain, lungs, liver, adrenal glands and lymph nodes were the most commonly involved sites with metastases.

### Overall Survival

**Fig. 1:**
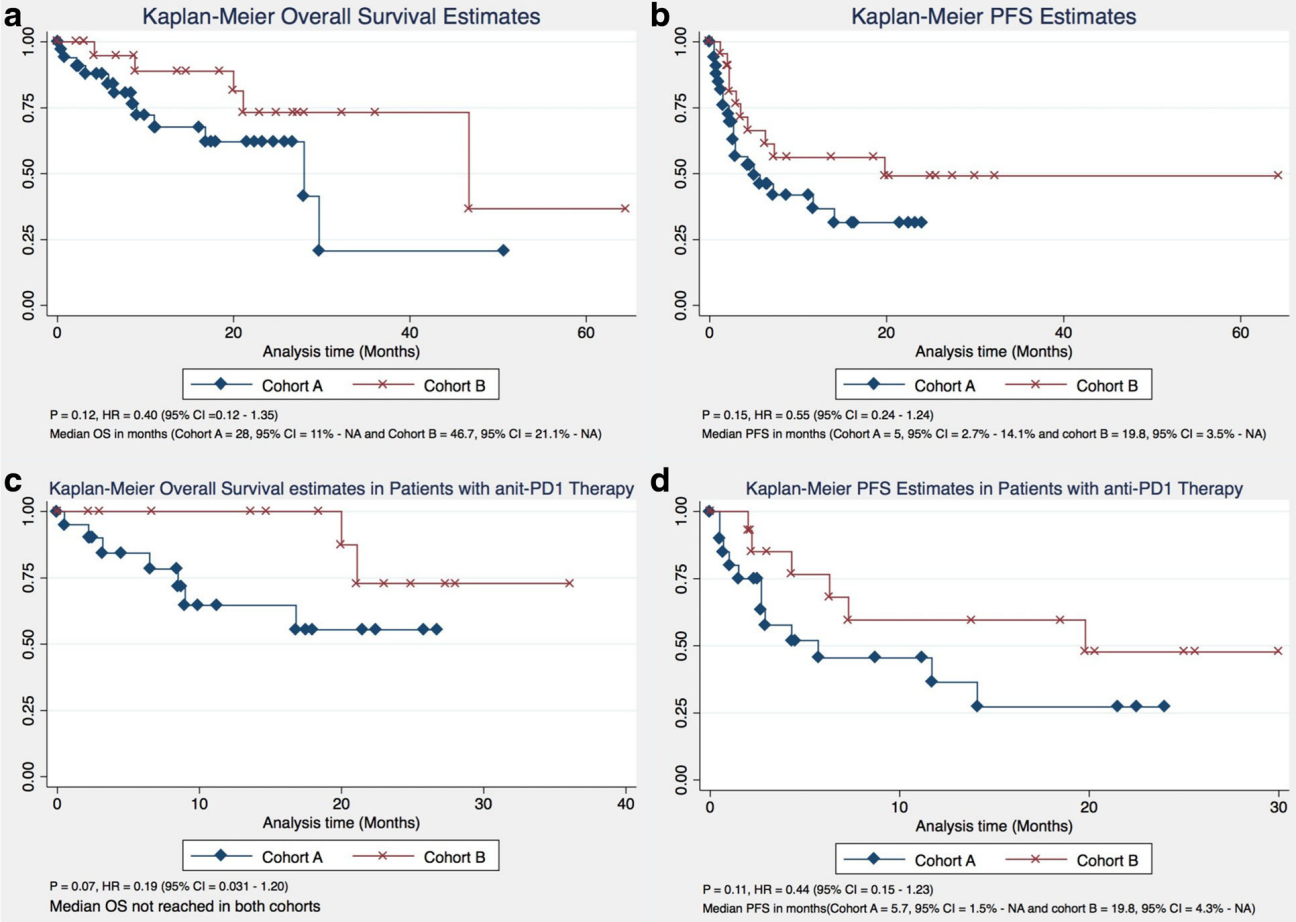
Overall and Progression Free Survival (**a**-**b**) and Overall and progression free survival in patients receiving PD-1 only therapy (**c**-**d**)

**Fig. 2:**
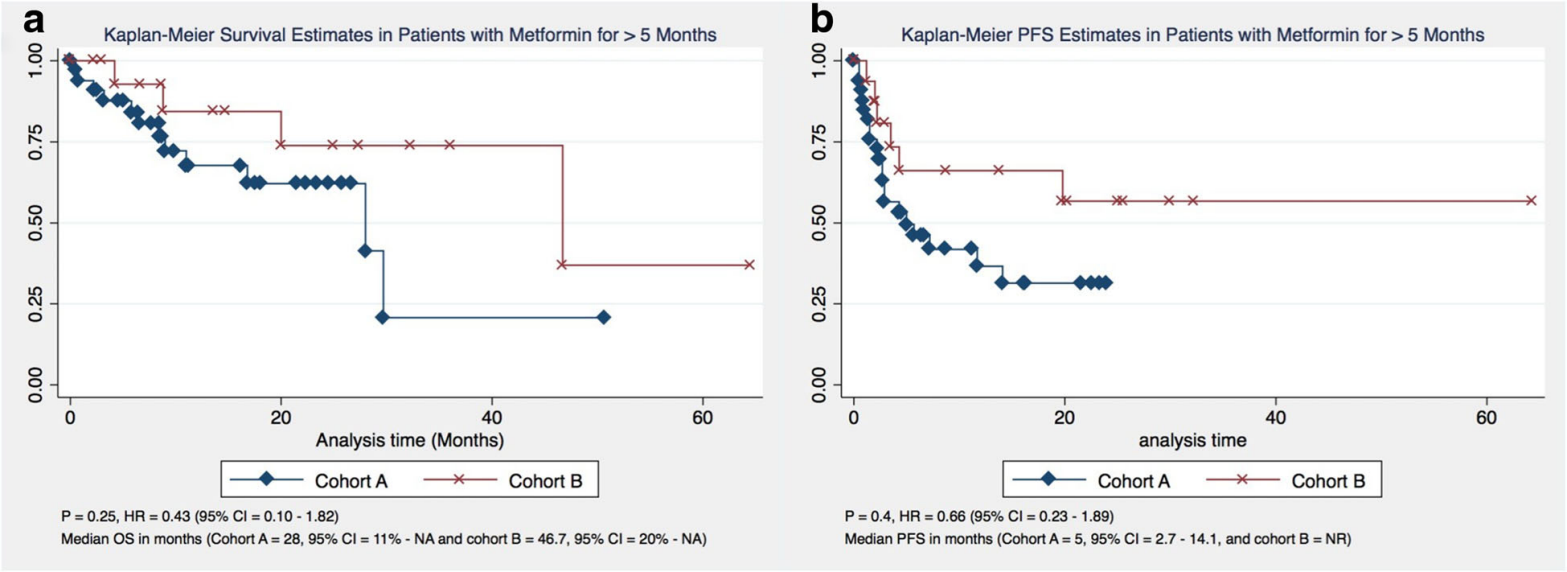
Overall and Progression Free Survival comparison with patients receiving metformin for > 5 months (**a**-**b**)

OS was calculated and compared between both cohorts using both the Kaplan-Meier estimate and multivariate analysis. Although OS was not significantly different between both cohorts [*P* = 0.12, HR 0.40 (95% CI = 0.12–1.35%)], the median OS was considerably longer in cohort B compared to cohort A (46.7 months vs. 28 months) (Fig. 1a). None of the confounders had any significant impact on OS. Furthermore, duration of metformin therapy had no impact on the OS (*P* = −.79, 95% CI = − 0.002 - 0.003%). On excluding patients who received metformin therapy for 5 months or less, there was no significant difference in OS between both cohorts either, and the median OS remained the same in both cohorts (Fig. 2a). Moreover, 88.2% of patients were alive by the end of the first year in cohort B compared to 67.7% of patients in cohort A. At the end of the third year, 73.3% of patients were alive in cohort B compared to only 20.7% in cohort A (Table 2).

**Table 2:** Overall and Progression Free Survival (All patients)

Cohort A (No Metformin)	Cohort B (Metformin)
Overall Survival	
• 67.7% patients alive at 1 year	• 88.2% patients alive at 1 year
• 62.0% patients alive at 2 years	• 73.3% patients alive at 2 years
• 20.7% patients alive at 3 years	• 73.3% patients alive at 3 years
Progression Free Survival	
• 36.6% patients free from progression at 1 year	• 56.1% patients free from progression at 1 year
• 31.3% patients free from progression at 2 years	• 49.1% patients free from progression at 2 years

On subset analysis, when patients were stratified based on anti-PD1 only therapy, no statistically significant difference in OS was found between both cohorts (*P* = 0.07, HR 0.19, 95% CI = 0.031–1.20%). None of the other variables have any significant impact on OS. Median OS was not reached in either cohort (Fig. 1c). At the end of first year, 100% of patients were alive in cohort B vs. 64.6% of patients in cohort A. Moreover, 72.9% patients were alive at the end of 2 years in cohort B compared to 55.4% of patients in cohort A (Table 3).

**Table 3:** Overall and Progression free survival in Patients receiving only PD-1 therapy

Cohort A (No Metformin)	Cohort B (Metformin)
Overall Survival	
• 64.6% patients alive at 1 year	• 100% patients alive at 1 year
• 55.4% patients alive at 2 years	• 72.9% patients alive at 2 years
Progression Free Survival	
• 36.3% patients free from progression at 1 year	• 59.6% patients free from progression at 1 year
• 27.2% patients free from progression at 2 years	• 47.6% patients free from progression at 2 years

### Progression free survival

A higher proportion of patients in cohort A were noted to have disease progression compared to cohort B [20 (60.6%) vs. 10 (45.45%), *P* = 0.26] (Table 1). PFS was not significantly different between both cohorts (*P* = 0.15, HR 0.55 (95% CI = 0.24–1.24%); however, median PFS was considerably longer in cohort B (19.8 months vs. 5 months) (Fig. 1b). On multivariate analysis, none of the confounders had any significant impact on PFS. Duration of metformin therapy had no significant impact PFS (*P* = 0.55, 95% CI = − 0.0091 - 0.0035%). In addition, 56.1% of patients were free from progression at the end of the first year in cohort B, compared to only 36.6% patients in cohort A. About 49.1% of patients were free from progression by the end of the second year in cohort B, compared to only 31.3% of patients in cohort A (Table 2).

When patients were stratified based on anti-PD-1 therapy only, no statistically significant difference in PFS was found between both cohorts (*P* = 0.11, HR 0.44, 95% CI = 0.15–1.23%) although the median PFS was longer in cohort B patients (19.8 months vs. 5.7 months) (Fig. 1d). On multi-variate analysis, patient gender was found to have a significant impact on PFS (*P* = 0.043, HR = 3.32, 95% CI = 1.03–10.65%). By the end of the first year, 59.6% of patients were free from progression in cohort B vs. only 36.3% of patients in cohort A. By the end of the 2nd year, 47.7% of patients were free from progression in cohort B compared to just 27.2% of patients in cohort A (Table 3).

### Best radiographic response

The best radiographic response was determined during treatment in both cohorts using the RECIST criteria v1.1. There was no statistically significant difference in BR in both cohorts (*P* = 0.39). A higher proportion of patients (27.7%) achieved CR in cohort B compared to 12.1% in cohort A. The ORR was also higher in cohort B compared to cohort A, although it was not statistically significant (68.2% vs. 54.5%, *P* = 0.31). Similarly, although the DCR was considerably higher in cohort B, it was not statistically significant either (77.3% vs. 60.6%, *P* = 0.19). The time to achieve BR was similar in both cohorts (2.7 vs. 2.8 months, *P* = 0.12) (Table 4).

**Table 4:** Overall Best Response Per RECIST V 1.1 and for Patients with Metformin Duration of > 5 Months (PR = Partial Response, CR = Complete Response, SD = Stable Disease, PD = Progressive Disease, ORR = Objective Response Rate, DCR = Disease Control Rate)

All Patients	No Metformin *N* = 33 (60%)	Metformin *N* = 22 (40%)	
Best Response			
PR	14 (42.4%)	9 (40.9%)	*P* = 0.39
CR	4 (12.1%)	6 (27.27%)	
SD	2 (6%)	2 (9.1%)	
PD	13 (39.4%)	5 (22.7%)	
ORR	54.5%	68.2%	*P* = 0.31
DCR	60.6%	77.3%	*P* = 0.19
Median Time to Achieve Best Response (months)	2.7	2.8	*P* = 0.12
Metformin Duration of > 5 Months	No Metformin *N* = 33 (67.3%)	Metformin *N* = 16 (32.7%)	
Best Response			
PR	14 (42.4%)	5 (31.2%)	*P* = 0.12
CR	4 (12.1%)	6 (37.5%)	
SD	2 (6%)	2 (12.5%)	
PD	13 (39.4%)	3 (18.8%)	
ORR	54.5%	68.7%	*P* = 0.34
DCR	60.6%	81.3%	*P* = 0.14
Median Time to Achieve Best Response (months)	2.7	3	*P* = 0.11

Patients were further stratified based on whether they received anti-PD-1 therapy or ipilimumab/nivolumab combination therapy. Among the patients receiving pembrolizumab, the ORR and DCR were higher in cohort B compared to cohort A without any statistical significance [(71.4% vs. 55%, *P* = 0.33) and (85.7% vs. 65%, *P* = 0.17) respectively]. Among patients who received ipilimumab/nivolumab combination therapy, patients in cohort A did relatively well as a higher number of patients achieved PR in cohort A (66.7% vs. 16.7%, *P* = 0.16). However, ORR and DCR were not statistically different between both cohorts (77.7% vs. 50%, *P* = 0.26 respectively for both ORR and DCR) (Table 5). It is noteworthy that the median duration of metformin therapy in patients who achieved CR in cohort B was 46.2 months compared to the overall median duration of metformin therapy of 13.5 months. However, on excluding the patients with metformin therapy of ≤ 5 months, the ORR remained at 68.7%, while the DCR increased to 81.3% (Table 4). Moreover, among patients with N-RAS mutations, ORR and DCR were 80% each in cohort A, and 75 and 100% respectively, in cohort B.

**Table 5:** Stratification of Best response based on type of Immunotherapy (PR = Partial Response, CR = Complete Response, SD = Stable Disease, PD = Progressive Disease, ORR = Objective Response Rate, DCR = Disease Control Rate)

Anti-PD-1	No Metformin *N* = 20 (60.6%)	Metformin *N* = 14 (63.6%)	
Best Response			
PR	8 (40%)	7 (50%)	*P* = 0.6
CR	3 (15%)	3 (21.4%)	
SD	2 (10%)	2 (14.3%)	
PD	7 (35%)	2 (14.3%)	
ORR	55%	71.4%	*P* = 0.33
DCR	65%	85.7%	*P* = 0.17
Median time to achieve best response (Months)	2.8	2.8	*P* = 0.28
Ipilimumab/Nivolumab	No Metformin *N* = 9 (27.3%)	Metformin *N* = 6 (27.3%)	
Best Response			
PR	6 (66.7%)	1 (16.7)	*P* = 0.16
CR	1 (11.1%)	2 (33.3%)	
SD	0 (0%)	0 (0%)	
PD	2 (22.2%)	3 (50%)	
ORR	77.7%	50%	*P* = 0.26
DCR	77.7%	50%	*P* = 0.26
Median time to achieve best response (Months)	2.5	2.8	*P* = 0.9

### Safety and tolerance

Both therapeutic categories were well tolerated in both cohorts, although there were some therapy-related side effects experienced by patients from both cohorts. Most of the patients had an ECOG-PS of 0–1 during therapy in both cohorts (*P* = 0.33) (Table 1). Fatigue was most commonly observed in patients from cohort A, [23 (69.7%) vs. 9 (40.1%), *P* = 0.034]. The overall proportion of patients developing immune-related adverse events were similar in both cohorts (60.6% vs. 59%, *P* = 0.9). Slightly more patients experienced a rash in cohort A [6 (18.2%) vs. 2 (9.1%), *P* = 0.34]. Two patients developed grade III-IV rash in cohort A, requiring prednisone therapy. A significantly higher proportion of patients had pneumonitis in cohort B [2 (6.1%) vs. 6 (27.3%), *P* = 0.02]. One patient in each cohort required hospitalization due to pneumonitis. Three (13.6%) patients had acute kidney injury in cohort B compared to 1 in cohort A (*P* = 0.13). A slightly higher proportion of patients had transaminitis in cohort A [7 (21.2%) vs. 4 (18.2%), *P* = 0.7]. Two patients in cohort B had grade IV transaminitis. Five patients in cohort A had grade III-IV transaminitis. All patients who developed transaminitis in both cohorts received steroid therapy. Six patients from cohort A and 5 patients from cohort B developed colitis (*P* = 0.6), and all of these patients except one required prednisone therapy. A higher proportion of patients were hospitalized due to treatment-related complications in cohort A [9 (27.3%) vs. 2 (9%), *P* = 0.1] compared to cohort B. Overall, the proportion of patients requiring prednisone therapy due to immune-related adverse events was similar between both cohorts (54.5% vs. 59%, *P* = 0.73) (Table 1).

## Discussion

Metformin is the most commonly used oral hypoglycemic agent from the biguanide class for the treatment of type-II Diabetes Mellitus [1]. Metformin inhibits protein synthesis and cell proliferation by regulating the LKB1/AMPL pathway [6]. Metformin also inhibits the UPR and activates the immune response against tumor cells [8]. In mouse models, it has been reported that the efficacy of the PD-1 blockage is potentiated by the metformin-induced reduction of tumor hypoxia [25]. Biguanides also inhibit the MDSCs and enhance the anti-tumor activity of the PD-1 blockade. Based on these findings derived from related literature, we performed a retrospective analysis of malignant melanoma patients who have received metformin in combination with ICIs and compared them with patients who have received ICIs only. To our knowledge, this is the first study of its kind.

The primary endpoint in our study is the ORR. A higher proportion of patients achieved an objective response in cohort B (68.2% vs. 54.5%); however, the difference was not significant (*P* = 0.31). Similarly, the proportion of patients who achieved disease control was higher is cohort B as well (77.3% vs. 60.6%, *P* = 0.19). The difference in ORRs was also considerable when further stratification based on anti-PD-1 therapy was done (71.4% vs. 55%, *P* = 0.33). Although these differences were not statistically significant, we believe that this was due to a very small sample size. In any case, there is an overall trend towards better outcomes in cohort B. In a study by Montaudié et al., none of the patients achieved either PR or CR at 6 months, whereas in this study, the median time to achieve the BR was 2.8 months. In this study, however, metformin monotherapy was used in patients who were previously on chemotherapy and/or ipilimumab [24]. It has been reported in the literature that patients with an N-RAS mutation respond well to immunotherapy [30]. Johnson et al. reported that patients with an N-RAS mutation have a 28% vs. 16% (*P* = 0.04) ORR to first-line therapy, whereas DCR was 50% vs. 20%, *P* = 0.07 [30]. In our study, 9 (20%) patients had an N-RAS mutation. ORR was 77.8% in N-RAS mutant patients compared to 63.3% in non-NRAS mutant patients (*P* = 0.42). DCR was also higher in N-RAS mutant patients (88.9% vs. 70%, *P* = 0.25). On stratifying patients based on N-RAS mutation, there was no significant difference in DCR and ORR between both cohorts.

The secondary endpoints in our study were OS and PFS. Again, due to small sample size, the differences in these endpoints were not statistically significant on both univariate and multivariate analyses, but we continued to observe the trend towards better outcomes in patients receiving metformin in combination with ICIs. The median OS was longer in cohort B patients compared to cohort A (46.7 vs. 28 months). The OS at the end of the first year was also higher in cohort B compared to cohort A (88.2% vs. 67.7%]. In a study by Montaudié et al., 72.6% patients were alive at 6 months [24]. In another study, the median OS was 16.1 vs. 16 months in a patient receiving BRAFV600E inhibitors with or without metformin [23]. PFS and OS were not significant either. In our study, the median PFS was also higher in cohort B (19.8 vs. 5 months) without any statistical significance. Furthermore, 56.1% of patients were free from disease progression at 1 year in cohort B compared to 36.6% patients in cohort A. These results are noteworthy compared to the results shown in other studies. In Montaudié et al.’s study, the median PFS was 1.6 months—considerably lower compared to the median PFS in our study [23]. No difference in OS and PFS were observed in both cohorts when stratified based on anti-PD-1 therapy, but the median PFS was again longer in cohort B, while the median OS was not reached in either cohort. It is also important to note that, on multivariate analysis in our study, gender had a significant impact on PFS when patients were stratified by anti-PD-1 therapy (*P* = 0.043, HR = 3.32, 95% CI = 1.03–10.65%). It appears that female patients tend to have better prognosis in malignant melanoma as reported by a few studies [31, 32]. In cohort A, the proportion of female patients was higher (42.4% vs. 31.8%). In a study reported by Schuchter et al., the 10-year survival rate was 86% in female patients compared to 68% in male patients [33]. Furthermore, a patient’s age has also been reported to be an independent prognostic factor in malignant melanoma [34]. It has been reported, moreover, that a 10-year increase in a patient’s age is associated with a decline in both 5-year and 10-year survival rates [35]. In our study, the mean age at the time of diagnosis was 60.8 years in cohort A vs. 67.4 years (*P* = 0.08) in cohort B. Though older, patients in cohort B showed a trend toward better outcomes.

We also included the patients receiving ipilimumab either alone or in combination with nivolumab in our study. Although mainly pre-clinical studies have evaluated the efficacy of PD-1 blockage with biguanides, a retrospective study evaluated the impact of different chronic medications on ipilimumab in malignant melanoma. In this study, 18% of patients achieved OR [*P* = 0.38, HR 0.49, 95% CI = 0.10–2.43%) [36]. A small proportion of patients received ipilimumab-only therapy in both cohorts of our study [4 (12.1%) vs. 2 (9.1%)], especially after FDA approval of nivolumab and pembrolizumab in 2014. Both patients achieved OR in cohort B; however, none of the patients achieved OR in cohort A.

In our study, although the difference in the primary and secondary endpoints were not statistically significant, there was a clear trend towards better outcomes in patients receiving metformin despite having almost similar characteristics (such as type of ICIs received, prior use of immunotherapies/ chemotherapies, mutation load, type of mutations, the TNM staging and the metastatic sites involved). Distribution of skeletal and visceral metastasis, and mean number of metastatic sites involved before therapy were also similar between both cohorts. However, cohort A had a higher proportion of female patients and contained a younger population, both of which are associated with better prognosis [31, 32, 34]. We further observed that the mean number of new metastatic sites appearing while on therapy of interest was significantly higher in cohort A (A:0.59 vs. B:1.51, *P* = 0.009). None of the prior studies have reported such observations.

The limitations of our study include small sample size, low power, retrospective nature and convenience sampling. We further did not exclude the patients who were shifted to another ICI in cohort B due to an already small sample size. Although if we exclude patients who have previously received any ICI in cohort B, the ORR and DCR in the remaining patients will be 77 and 93% respectively. These numbers are still significantly greater compared to that of cohort A.

## Conclusion

We observed favorable treatment-related outcomes (ORR, DCR, OS, and PFS) in patients who have received metformin in combination with ICIs without reaching significance due, probably, to the small sample size. Metformin has been shown to reduce cancer related mortality in solid tumors in a retrospective review [8]. Pre-clinical studies have shown a beneficial effect of metformin on the malignant melanoma models as well. However, previous clinical studies have not demonstrated a significant impact of metformin on malignant melanoma when used alone or in combination. To our knowledge, no other study has been reported to evaluate the effect of metformin in combination with ICIs in malignant melanoma. However, before we can recommend the use of metformin as a conventional additive therapy for malignant melanoma in a combination of other ICIs regardless of patient’s diabetic status, larger prospective clinical studies will need to be done. Prospective randomized clinical trials are underway to evaluate the clinical benefit of combining metformin with BRAF/MEK kinase inhibitors (NCT01638676 and NCT02143050) [37, 38] but to our knowledge, no such trial is being conducted to evaluate the efficacy of metformin with ICIs especially anti-PD-1 combination.

## Abbreviations

BR: Best Response
CR: Complete Response
CTLA-4: Cytotoxic T-Lymphocyte-Associated Protein 4
DCR: Disease Control Rate
IAE: Immune-related Adverse Events
ICI: Immune Checkpoint Inhibitors
ORR: Objective Response Rate
OS: Overall Survival
PD: Progressive Disease
PD-1: Programmed Cell Death Protein 1
PFS: Progression-free Survival
PR: Partial Response
RECIST: Response Evaluation Criteria in Solid Tumors
SD: Stable Disease

## References

[1] Dardano A, Penno G, Prato SD, Miccoli R (2014). Optimal therapy of type 2 diabetes: a controversial challenge. Aging.

[2] Rang HP (2003). Pharmacology.

[3] Zhou G, Myers R, Li Y (2001). Role of AMP activated protein kinase in mechanism of metformin action. J Clin Invest.

[4] Mu J, Brozinick JT, Valladares O (2001). A role for AMP-activated protein kinase in con- traction- and hypoxia-regulated glucose transport in skeletal muscle. Mol Cell.

[5] Fryer LG, Parbu-Patel A, Carling D (2002). The anti-diabetic drugs rosiglitazone and metformin stimulate AMP-activated protein kinase through distinct signaling pathways. J Biol Chem.

[6] Sahra IB, Marchand-Brustel YL, Tanti J, Bost F (2010). Metformin in Cancer therapy: a new perspective for an old Antidiabetic drug?. Mol Cancer Ther.

[7] Zakikhani M, Dowling R, Fantus IG (2006). Metformin is an AMP kinase-dependent growth inhibitor for breast cancer cells. Cancer Res.

[8] Franciosi M, Lucisano G, Lapice E (2013). Metformin therapy and risk of Cancer in patients with type 2 diabetes: systematic review. PLoS One.

[9] Cerezo M, Tomic T, Ballotti R, Rocchi S (2014). Is it time to test biguanide metformin in the treatment of melanoma?. Pigment Cell Melanoma Res.

[10] Gwinn DM, Shackelford DB, Egan DF (2008). AMPK phosphorylation of raptor mediates a metabolic checkpoint. Mol Cell.

[11] Buzzai M, Jones RG, Amaravadi RK (2007). Systemic treatment with the antidiabetic drug metformin selectively impairs p53-deficient tumor cell growth. Cancer Res.

[12] Algire C, Amrein L, Zakikhani M, Panasci L, Pollak M (2010). Metformin blocks the stimulative effect of a high-energy diet on colon carcinoma growth in vivo and is associated with reduced expression of fatty acid synthase. Endocr Relat Cancer.

[13] Vallianou NG, Evangelopoulos A, Kazazis C (2013). Metformin and Cancer. Rev Diabet Stud.

[14] Song CW, Lee H, Dings RPM (2012). Metformin kills and radiosensitizes cancer cells and preferentially kills cancer stem cells. Sci Rep.

[15] SEER Stat Fact Sheets: Melanoma of the Skin. Available at: https://seer.cancer.gov/statfacts/html/melan.html. Retrieved on 6/13/2018.

[16] Thiery JP, Sleeman JP (2006). Complex networks orchestrate epithelial- mesenchymal transitions. Nat Rev Mol Cell Biol.

[17] Nakamura M, Tokura Y (2011). Epithelial-mesenchymal transition in the skin. J Dermatol Sci.

[18] Cerezo M, Tichet M, Abbe P (2013). Metformin blocks melanoma invasion and metastasis development in AMPK/p53-dependent manner. Mol Cancer Ther.

[19] Tomic T, Botton T, Cerezo M (2011). Metformin inhibits melanoma development through autophagy and apoptosis mechanisms. Cell Death Dis.

[20] Smalley KS (2010). PLX-4032, a small-molecule B-Raf inhibitor for the potential treatment of malignant melanoma. Curr Opin Investig Drugs.

[21] Russell WJ, Barbie DA, Flaherty KT (2018). Mechanisms of resistance to immune checkpoint inhibitors. Br J Cancer.

[22] Aplin AE, Kaplan FM, Shao Y (2011). Mechanisms of resistance to RAF inhibitors in melanoma. J Invest Dermatol.

[23] Livingstone E, Swann S, Lilla C, Schadendorf D, Roesch A (2015). Combining BRAFV600E inhibition with modulators of the mitochondrial bioenergy metabolism to overcome drug resistance in metastatic melanoma. Exp Dermatol.

[24] Montaudié H, Cerezo M, Bahadoran P (2017). Metformin monotherapy in melanoma: a pilot, open-label, prospective, and multicentric study indicates no benefit. Pigment Cell & Melanoma Research.

[25] Scharping NE, Menk AV, Whetstone RD (2016). Efficacy of PD-1 blockade is potentiated by metformin-induced reduction of tumor hypoxia. Cancer Immunol Res.

[26] Kim SH, Man L, Trousil S (2017). Phenformin inhibits myeloid-derived suppressor cells and enhances the anti-tumor activity of PD-1 blockade in melanoma. J Investig Dermatol.

[27] Marvel D, Gabrilovich DI (2015). Myeloid-derived suppressor cells in the tumor micro- environment: expect the unexpected. J Clin Invest.

[28] Eisenhauer E, Therasse P, Bogaerts J, Shankar L (2008). 32 INVITED new response evaluation criteria in solid tumors: revised RECIST guideline version 1.1. Eur J Cancer Suppl.

[29] Stat Trek: Teach Yourself Statistics. Available at: http://stattrek.com/statistics/random-number-generator.aspx. Retrieved on 3/17/2018.

[30] Johnson DB, Lovly CM, Flavin M (2015). Impact of NRAS mutations for patients with advanced melanoma treated with immune therapies. Cancer Immunol Res.

[31] Masback A, Olsson H, Westerdahl J (2001). Prognostic factors in invasive cutaneous malignant melanoma: a population-based study and review. Melanoma Res.

[32] Vossaert KA, Silverman MK, Kopf AW (1992). Influence of gender on survival in patients with stage I malignant melanoma. J Am Acad Dermatol.

[33] Schuchter L, Schultz DJ, Synnestvedt M (1996). A prognostic model for predicting 10-year survival in patients with primary melanoma. The Pigmented Lesion Group. Ann Intern Med.

[34] Chang CK, Jacobs IA, Vizgirda VM (2003). Melanoma in the elderly patient. Arch Surg.

[35] Balch CM, Soong SJ, Gershenwald JE (2001). Prognostic factors analysis of 17,600 melanoma patients: validation of the American joint committee on Cancer melanoma staging system. J Clin Oncol.

[36] Failing JJ, Finnes HD, Kottschade LA, Allred JB, Markovic SN (2016). Effects of commonly used chronic medications on the outcomes of ipilimumab therapy in patients with metastatic melanoma. Melanoma Res.

[37] A Phase I/II Trial of Vemurafenib and Metformin to Melanoma Patients - Full Text View. Full Text View - ClinicalTrials.Gov, clinicaltrials.gov/ct2/show/NCT01638676.

[38] Study of Dabrafenib, Trametinib and Metformin for Melanoma Patients - Full Text View. Full Text View - ClinicalTrials.Gov, clinicaltrials.gov/ct2/show/NCT02143050.

